# The Effects of Annatto Tocotrienol on Bone Biomechanical Strength and Bone Calcium Content in an Animal Model of Osteoporosis Due to Testosterone Deficiency

**DOI:** 10.3390/nu8120808

**Published:** 2016-12-14

**Authors:** Kok-Yong Chin, Dhivakaran Gengatharan, Fadlin Sakina Mohd Nasru, Rehan Amalia Khairussam, Sherlyn Lai Hui Ern, Siti Aina Wahidah Aminuddin, Soelaiman Ima-Nirwana

**Affiliations:** Department of Pharmacology, Faculty of Medicine, Universiti Kebangsaan Malaysia Medical Centre, Cheras 56000, Malaysia; dhiva09@gmail.com (D.G.); fadlinsakina@yahoo.com (F.S.M.N.); rehanamaliakhairussam@gmail.com (R.A.K.); sherlynlaihe@gmail.com (S.L.H.E.); Aina_aminuddin93@yahoo.com (S.A.W.A.); imasoel@ppukm.ukm.edu.my (S.I.-N.)

**Keywords:** antioxidant, calcium, orchidectomy, phosphate, vitamin E

## Abstract

Osteoporosis reduces the skeletal strength and increases the risk for fracture. It is an underdiagnosed disease in men. Annatto tocotrienol has been shown to improve bone structural indices and increase expression of bone formation genes in orchidectomized rats. This study aimed to evaluate the effects of annatto tocotrienol on biomechanical strength and calcium content of the bone in orchidectomized rats. Thirty three-month-old male Sprague-Dawley rats were randomly assigned to five groups. The baseline control (BC) group was sacrificed at the onset of the study. The sham-operated group (SHAM) received olive oil (the vehicle of tocotrienol) orally daily and peanut oil (the vehicle of testosterone) intramuscularly weekly. The remaining rats were orchidectomized and treated with three different regimens, i.e., (1) daily oral olive oil plus weekly intramuscular peanut oil injection; (2) daily oral annatto tocotrienol at 60 mg/kg plus weekly intramuscular peanut oil injection; (3) daily oral olive oil plus weekly intramuscular testosterone enanthate injection at 7 mg/kg. Blood, femur and tibia of the rats were harvested at the end of the two-month treatment period for the evaluation of serum total calcium and inorganic phosphate levels, bone biomechanical strength test and bone calcium content. Annatto-tocotrienol treatment improved serum calcium level and tibial calcium content (*p* < 0.05) but it did not affect femoral biomechanical strength (*p* > 0.05). In conclusion, annatto-tocotrienol at 60 mg/kg augments bone calcium level by preventing calcium mobilization into the circulation. A longer treatment period is needed for annatto tocotrienol to exert its effects on bone strength.

## 1. Introduction

Osteoporosis is characterized by a reduction in bone density and quality, ultimately leading to reduced bone strength and increased fracture risk, particularly at the hip, spine and wrist [1]. The prevalence of osteoporosis is rising concurrently with the increase in life span of the elderly population globally. Estimation in the year 2000 revealed that nine million osteoporotic fractures occurred worldwide [2]. The European countries contributed to the highest number of cases and the most number of disability adjusted life years lost due to osteoporotic fractures [2]. However, projection shows that by the year 2050, 50% of osteoporotic hip fractures will occur in Asia [3]. Osteoporosis is more prevalent in women compared to men [2]. Despite this, male osteoporotic fracture patients suffer from greater rate of mortality and second fracture compared to their female counterpart [4,5]. This shows that male osteoporotic patients remain underdiagnosed and undertreated [6,7].

Age-related bone loss in men transpires concurrently with the decline of bioavailable and free fraction of testosterone [8,9,10] This phenomenon, also known as late-onset hypogonadism or testosterone deficiency syndrome [11], is caused by the increased sex hormone-binding globulin (SHBG) level with age, which limits the bioavailability of testosterone to body tissues like bone [12]. Testosterone deficiency exerts a direct impact and indirect impact via conversion to estrogen on cells governing bone remodeling [13]. Apart from that, profound testosterone deficiency has been shown to affect calcium metabolism by increasing excretion of calcium, predominantly from the skeletal compartment [14]. The effects of testosterone deficiency and supplementation on calcium balance are so significant that it can be reflected in circulating levels of calcium [15]. This phenomenon may be mediated by increased parathyroid hormone triggered by testosterone deficiency [15]. Male osteoporosis due to testosterone deficiency is treated with testosterone replacement therapy, but the recipients are at risk for prostate cancer and hyperplasia, and polycythemia [16]. Alternative treatments effective in increasing bone mineral density, such as bisphosphonates and teriparatide, are available, but they come with a range of undesirable side effects [17,18]. This has sparked the interest to find alternative therapies for male osteoporosis.

Tocotrienol, a subfamily of vitamin E, is one of the potential antiosteoporotic agents being studied [19,20,21]. Tocotrienol can be further divided into four homologues, namely alpha-, beta-, gamma- and delta-tocotrienol [22]. Tocotrienol can be found in various food sources, particularly rice bran, palm oil, and annatto oil [22]. Tocotrienol derived from annatto seed is unique because it contains 90% delta-tocotrienol, 10% gamma-tocotrienol and no tocopherols, another subfamily of vitamin E [23]. Alpha-tocopherol may be detrimental to the bone because it promotes osteoclast fusion and increases bone resorption in normal young rats [24]. This property is not seen with other vitamin E isomers [24]. It also competes with other vitamin E isomers for alpha-tocopherol transfer protein (αTTP), resulting in decreased secretion of other vitamin E isomers into lipoproteins and decreased delivery to tissues [25]. 

Annatto tocotrienol was previously demonstrated to prevent bone loss in gonadectomized rats [26,27,28,29]. It could prevent the deterioration of bone structure, and increase mineralizing surface, osteoblast number and osteoid volume in orchidectomized male rats [28,29]. These anabolic effects might be exerted through increased expression of bone formation genes [29]. Similar bone protective effects of annatto tocotrienol were observed in ovariectomized rats [26,27]. In addition, annatto tocotrienol also augmented the bone biomechanical strength of the ovariectomized rats [27], but this has not been determined in orchidectomized rats so far. A previous study by Ima-Nirwana et al. showed that palm vitamin E at 60 mg/kg for eight months could increase bone calcium content in orchidectomized rats [30], yet there was no data showing that annatto tocotrienol could achieve similar effects. 

This study aimed to determine the effects of annatto tocotrienol on bone calcium content and bone biomechanical parameters in an orchidectomized male rat model. Considering the positive evidence primarily derived from bone histomorphometry [28,29], we hypothesized that annatto tocotrienol could improve bone calcium content and biomechanical strength in orchidectomized rats in eight weeks. In bridging the knowledge gap, we hope to validate the use of annatto tocotrienol as an effective antiosteoporotic agent in men.

## 2. Materials and Methods 

### 2.1. Animals and Treatment 

Annatto tocotrienol consisting of 90% delta-tocotrienol and 10% gamma-tocotrienol was a gift from American River Nutrition (Hadley, MA, USA). It was diluted in olive oil (Bartolini Emilio, Arrone Terni, Italy) and administered orally at a dose of 60 mg/kg body weight. Testosterone enanthate (Jesalis Pharma, Jena, Germany) was diluted with peanut oil (Sime Darby, Subang Jaya, Malaysia) and administered intramuscularly at a dose of 7 mg/kg body weight. 

Thirty three-month-old male Sprague-Dawley rats were obtained from the Laboratory Animal Resource Unit, Universiti Kebangsaan Malaysia, Kuala Lumpur, Malaysia. The rats were housed individually in plastic cages and provided with free access to tap water and standard rat chow (Gold Coin, Port Klang, Malaysia) under room temperature and natural light/dark cycle. Following a week of acclimatization, the rats were randomized into five groups of six rats, namely baseline control (BC), sham-operated (SH), orchidectomized control (ORCH), annatto tocotrienol-treated (AnTT), and testosterone enanthate-treated (TE) groups. The baseline control (BC) group was sacrificed at the onset of the study. The ORCH, AnTT and TE groups underwent bilateral orchidectomy. The SH group was subjected to similar surgical stress, but their testes were retained. The AnTT group was treated with oral annatto tocotrienol 60 mg/kg daily while the SH, ORCH and TE groups were given equal volume of olive oil orally daily. The TE group received intramuscular testosterone enanthate at 7 mg/kg weekly while the other groups received equal volume of intramuscular peanut oil injection weekly. After eight weeks of treatment, all rats were sacrificed and their bones were harvested and kept under −70 °C for biomechanical strength test and calcium content assay.

### 2.2. Biochemical Analysis

Blood of the rats was collected at the end of the treatment period prior to euthanasia. Serum was extracted immediately after centrifuging the blood at 3000 rpm for 10 min at 4 °C. The serum was stored at −70 °C until analysis. Calcium and phosphate were measured using the QuantiChrom™ Calcium and Phosphate Assay Kit respectively (BioAssay Systems, Hayward, NY, USA) based on calorimetric method. 

### 2.3. Bone Biomechanical Strength Test 

**Figure 1:**
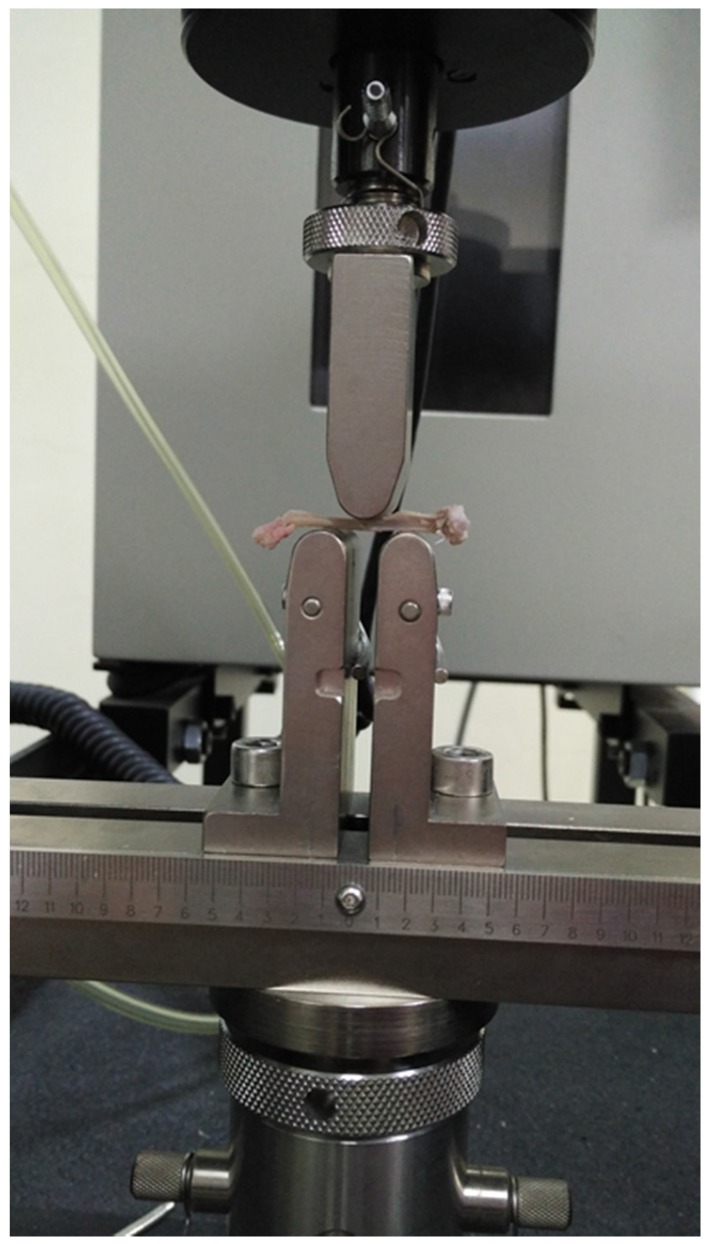
Biomechanical strength test on the femur.

The biomechanical strength test was conducted using Instron Universal Testing Machine (5560 Instron, Canton, OH, USA) (Figure 1) with Bluehill 2 software package (Instron, Canton, OH, USA). Briefly, the right femur cleaned of soft tissue was kept wet by phosphate-buffered saline socked gauze before the testing. It was mounted on two inferior supports and a load (speed 5 mm/min; span length 10 mm) was applied to the midshalf on its anterior surface until fractured. The data was then analyzed by Bluehill software to calculate load (N), strain (MPa), stress (mm/mm) and extension (MPa) of the bone. 

### 2.4. Bone Calcium Content 

The right tibias, cleaned of soft tissues, were dried in an oven at 100 °C for 24 h. They were then ashed in a furnace at 800 °C for 12 h. The ash was weighed and dissolved in 3 mL of nitric acid and later diluted in lanthanum chloride. Calcium chloride was measured with an Atomic Absorption Spectrophotometer (Shimadzu AA-680, Shimadzu, Kyoto, Japan) at 422.7 nm. 

### 2.5. Statistical Analysis 

Data analysis was performed using the Statistical package for Social Sciences software version 20.0 (IBM, Armonk, NY, USA). The Shapiro-Wilk test was used to assess the normality of the data. Normally distributed variables were analyzed by using analysis of variance (ANOVA) followed by Tukey’s post-hoc test. Skewed data were analyzed using Kruskal-Wallis test and Mann-Whitney U-test as a post-hoc pairwise comparison. The statistical differences were assumed significant at *p* < 0.05. The results were expressed as mean values ± standard error of the mean (SEM).

## 3. Results

**Figure 2:**
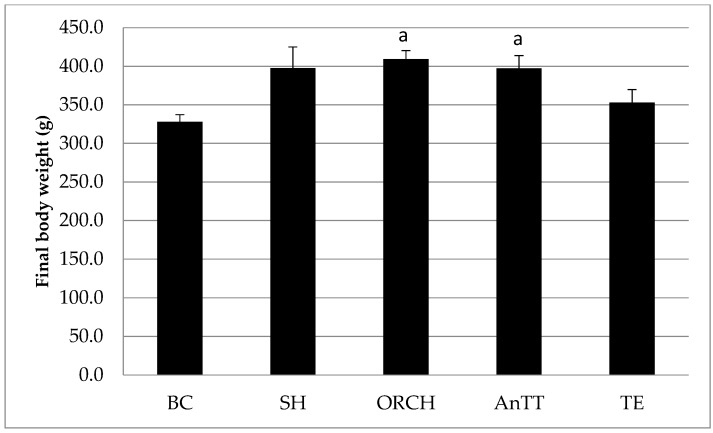
Final body weight of the rats. The data are shown as mean with standard error of the mean. Letter ‘a’ indicates significant difference versus the baseline group. Abbreviation: AnTT, annatto tocotrienol-supplemented group; BC, baseline control group; ORCH, orchidectomized group; SH, sham-operated; TE, testosterone enanthate-supplemented group.

The final body weight among the treatment groups was similar, with the exception that ORCH and AnTT groups were significantly heavier than BC group (*p* < 0.05) (Figure 2). 

**Figure 3:**
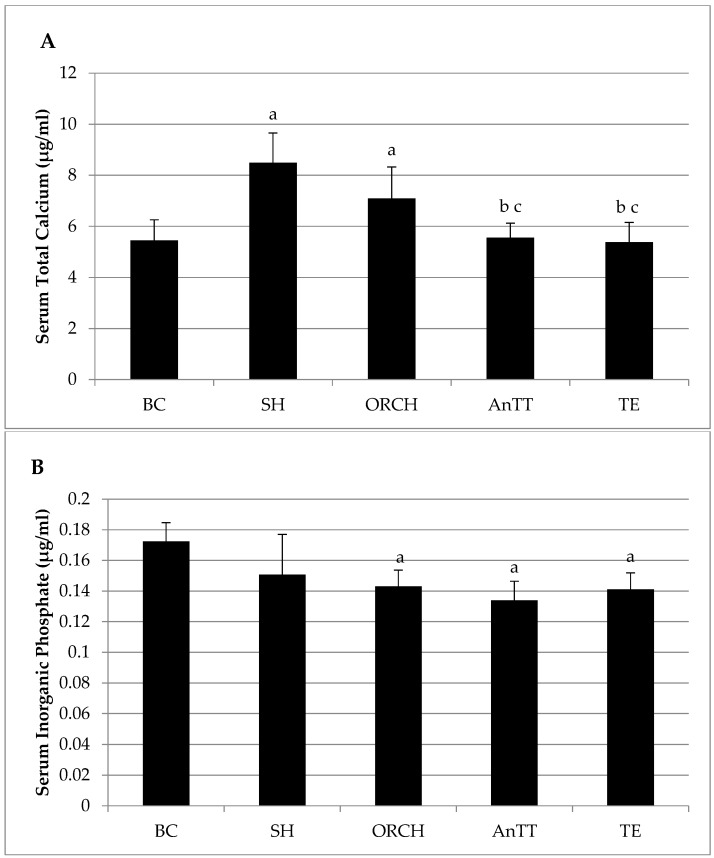
Post-treatment serum total calcium (**A**) and inorganic phosphate level (**B**) in rats. The data are shown as mean with standard error of the mean. Letter ‘a’ indicates significant difference versus the baseline group; ‘b’ versus the sham-operated group; ‘c’ versus the orchidectomized group. Abbreviation: AnTT, annatto tocotrienol-supplemented group; BC, baseline control group; ORCH, orchidectomized group; SH, sham-operated; TE, testosterone enanthate-supplemented group.

There was no significant difference in serum total calcium level between SH and ORCH group (*p* > 0.05). Serum total calcium level was significantly lower in AnTT and TE group compared to ORCH and SH group (*p* < 0.05) (Figure 3A). No significant difference was detected in serum inorganic phosphate level among SH, ORCH, AnTT and TE groups (*p* > 0.05) (Figure 3B). 

**Figure 4:**
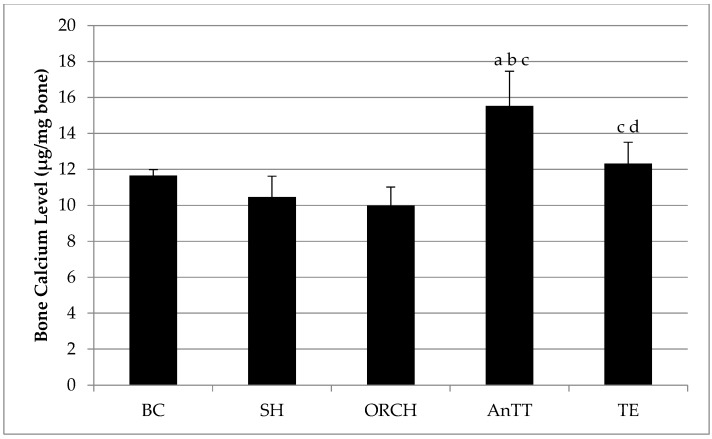
Post-treatment bone calcium level in rats. The data are shown as mean with standard error of the mean. Letter ‘a’ indicates significant difference versus the baseline group; ‘b’ versus the sham-operated group; ‘c’ versus the orchidectomized group; ‘d’ versus the annatto tocotrienol-supplemented group. Abbreviation: AnTT, annatto tocotrienol-supplemented group; BC, baseline control group; ORCH, orchidectomized group; SH, sham-operated; TE, testosterone enanthate-supplemented group.

Bone calcium level was not significantly different between the SH and ORCH group (*p* < 0.05). Both AnTT and TE group had significantly higher bone calcium level compared to ORCH group (*p* < 0.05). Annatto tocotrienol treatment increased the calcium content more than testosterone enanthate (*p* < 0.05) (Figure 4). 

**Figure 5:**
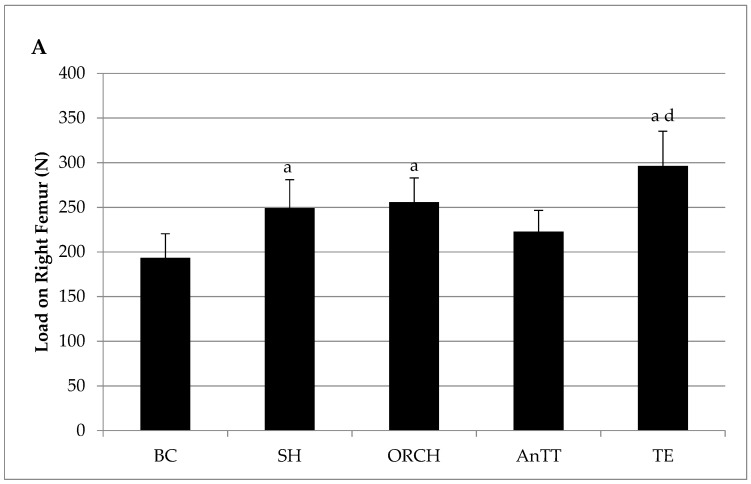

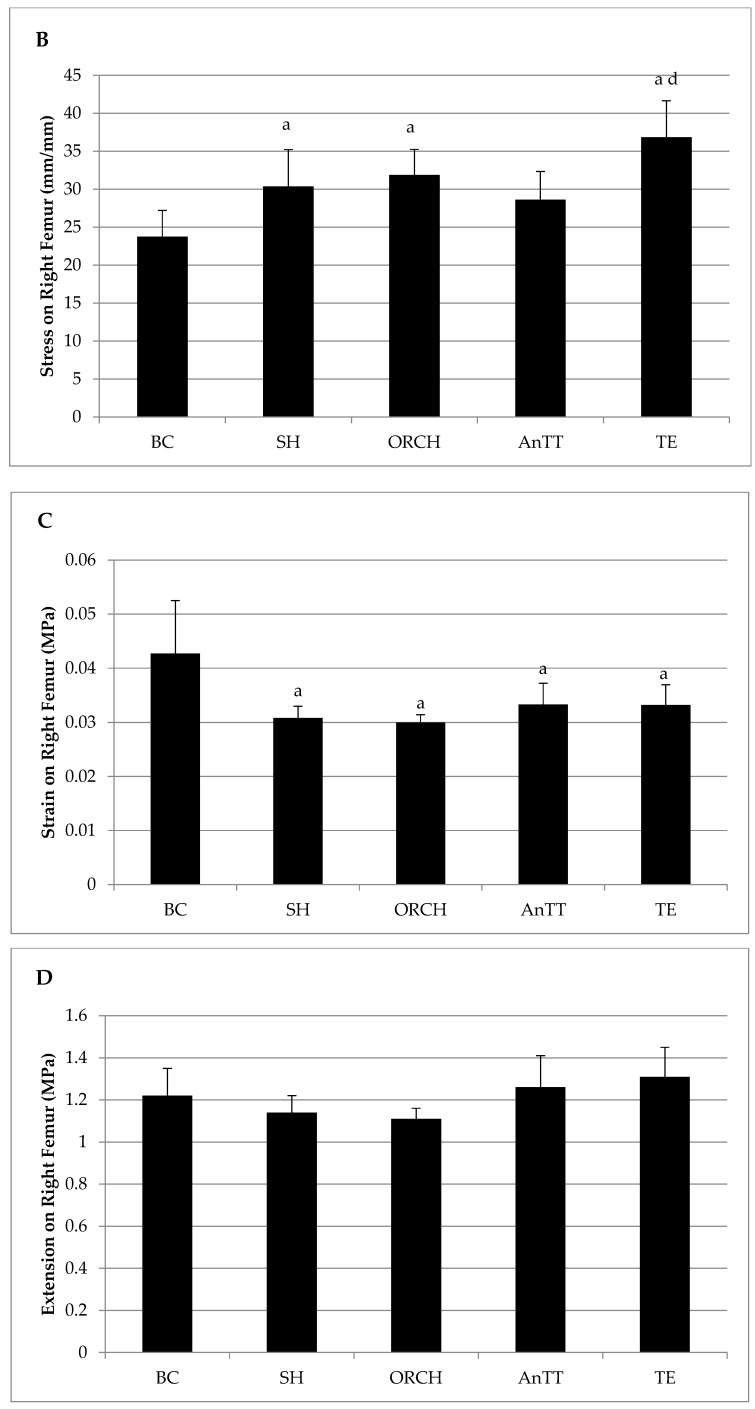
Post-treatment bone biomechanical strength parameters of the right femur, i.e., load (**A**); stress (**B**); strain (**C**) and extension (**D**). The data are shown as mean with standard error of the mean. Letter ‘a’ indicates significant difference versus the baseline group; ‘d’ versus the annatto tocotrienol-supplemented group. Abbreviation: AnTT, annatto tocotrienol-supplemented group; BC, baseline control group; ORCH, orchidectomized group; SH, sham-operated; TE, testosterone enanthate-supplemented group.

There were no significant changes in biomechanical strength indices between SH and ORCH groups (*p* > 0.05). Supplementation with annatto tocotrienol did not improve the biomechanical strength of the femur in rats (*p* > 0.05). Load, stress and strain of the rats treated with testosterone were significantly higher compared to the AnTT group (*p* < 0.05). No significant difference was found in the extension of the femur among the study groups (*p* > 0.05) (Figure 5A–D). 

## 4. Discussion

The present study showed that testosterone deficiency did not cause significant changes in circulating total calcium and inorganic phosphate levels, bone calcium content and bone biomechanical strength (*p* < 0.05). Annatto tocotrienol suppressed serum total calcium level and increased femoral calcium content in orchidectomized rats (*p* < 0.05). However, these changes did not translate to an augmented bone biomechanical strength in the annatto tocotrienol-supplemented rats (*p* > 0.05). Testosterone exerted similar effects as tocotrienol on serum total calcium level and bone calcium content (*p* < 0.05). The femoral load, stress and strain of testosterone-treated rats were better than annatto tocotrienol-treated rats (*p* < 0.05).

Testosterone deficiency did not induce significant alteration in serum calcium level in male rats. This observation is supported by a previous study showing that testosterone played a minor role compared to estrogen in calcium regulation in men [31]. In that study, men were deprived of both testosterone and estrogen, and estrogen experienced an increase in serum calcium level compared to baseline, but the serum calcium level in men deprived of testosterone did not change significantly [31]. In another study, high-dose testosterone enanthate supplementation for 16–20 weeks in a group of healthy young men lowered their serum calcium level significantly without changing their calcium excretion pattern [32]. Therefore, calcium level is affected by high-dose exogenous testosterone exposure, but not by variation in endogenous testosterone. 

The studies of annatto tocotrienol on serum calcium and phosphate levels, bone mineral content and bone mechanical strength in the testosterone deficiency model are limited. Therefore, comparison was made with studies using other models of bone loss and tocotrienol derived from other sources in the following discussion. In this study, both annatto tocotrienol and testosterone significantly lowered the serum total calcium level, presumably by preventing mobilization of skeletal calcium into the circulation. Decreased mobilization of calcium reserve caused by annatto tocotrienol was evidenced by higher femoral calcium content in the AnTT group. This was consistent with the study by Ima Nirwana et al., which demonstrated that palm vitamin E 60 mg/kg for eight months preserved femoral and vertebral calcium content in orchidectomized rats [30]. On the other hand, Muhammad et al. showed that palm tocotrienol treatment at 60 mg/kg for eight weeks did not increase lumbar calcium content in ovariectomized rats [33]. Extended treatment of palm vitamin E at 30 mg/kg and 60 mg/kg for 10 months also failed to augment lumbar and femoral calcium level in ovariectomized rats [34]. Thus, there might be gender differences in the skeletal protective effects of tocotrienol, whereby it is more effective in preserving calcium in male rats.

The calcium conserving effects of tocotrienol have been implied by many previous studies. Norazlina et al. indicated that vitamin E was essential for normal calcium metabolism. Rats receiving vitamin E-deficient diet suffered from increased parathyroid level, impaired calcium absorption and low calcium content in the lumbar spine [35,36]. Supplementation with gamma-tocotrienol could preserve the calcium content in rats fed with vitamin E-deficient diet [37].

Osteoclasts are the bone cells responsible for bone resorption [38]. Thus, reducing osteoclastic activity could prevent bone resorption that releases calcium from the bone into the circulation. Chin et al. showed that supplementation of annatto tocotrienol at 60 mg/kg decreased the osteoclast number and eroded surface on the trabecular bone of orchidectomized rats [28]. Similar observations were also obtained in studies supplementing annatto tocotrienol or palm tocotrienol in estrogen-deficient rats [26]. In vivo studies also demonstrated that individual isomers of tocotrienol could prevent the formation of osteoclast-like cells and inhibit their resorption activity [39]. The reduction in osteoclastic activity caused by annatto tocotrienol could contribute to lower circulating calcium level in the rats. However, the circulating phosphate level was not altered significantly in rats receiving tocotrienol treatment. Circulating phosphate level might be regulated more tightly, thus its variations could be less apparent. Besides, the breakdown of hydroxyapatite, the predominant form of calcium storage in the bone, yields five calcium ions and three phosphate ions. Thus, variation in bone resorption activity might give rise to greater changes in calcium level than in phosphate level. Besides, improvement in bone calcium content in the rats treated with annatto tocotrienol might be contributed by enhanced bone formation activity. Chin et al. demonstrated that annatto tocotrienol at 60 mg/kg for eight weeks specifically increased the expression of bone formation genes coding for alkaline phosphatase, collagen type I alpha 1, osteopontin and beta-catenin in orchidectomized rats [29]. In an osteopenic model due to estrogen deficiency, palm tocotrienol at 60 mg/kg for two months increased the mineral apposition rate and bone formation rate in rats [40,41]. Gamma-tocotrienol and palm tocotrienol-rich fraction at 60 mg/kg for two months was able to exert similar actions in a bone loss model due to nicotine in male rats [42]. Moreover, previous studies showed that annatto-tocotrienol supplementation could increase osteoblast number and osteoid volume in gonadectomized rats [26,28]. These studies showed that both annatto and palm tocotrienol could increase osteoblastic proliferation, survival and activity in osteopenic rats, thus contributing to increased mineralization and calcium storage in the bone. 

Material and geometric properties are the main contributors of the skeletal biomechanical strength [43]. Yarrow et al. showed that orchidectomy did not significantly reduce the load and stiffness of the bone in male rats [44]. The enhanced bone calcium content caused by annatto tocotrienol should have improved the material properties of the bone, but it did not translate to better biomechanical indices in the AnTT group. It was speculated that the geometrical properties of the bone were not altered in eight weeks by annatto tocotrienol supplementation. This was supported by a previous observation that supplementation of annatto tocotrienol at 60 mg/kg for eight weeks did not improve the structural model index, which is an assessment of geometrical structure of trabecular bone generated using micro-computed tomography, in orchidectomized rats [29]. Using an estrogen-deficient bone loss model, Muhammad et al. and Nazrun et al. showed that palm tocotrienol at 60 mg/kg for two months did not increase the biomechanical strength of osteopenic rats [33,45]. However, Shuid et al. showed that gamma-tocotrienol at 60 mg/kg for two months was able to increase biomechanical strength of the femur in normal male rats [46]. We hypothesize that tocotrienol might need a longer time to augment bone biomechanical strength in rats deficient in sex hormone compared to normal rats. On the other hand, bone biomechanical strength was better in rats treated with testosterone compared to rats treated with annatto tocotrienol. This was consistent with the previous findings that improvements in bone structural parameters were better in testosterone-treated rats compared to annatto treated rats [29]. Despite the lack of difference in bone strength between rats with and without testosterone deficiency, Yarrow et al. demonstrated that testosterone enanthate at 7 mg/kg could improve load but not stiffness of the bone [44]. 

Several limitations need to be addressed in this study. The study duration was eight weeks, which might be insufficient to augment the bone biomechanical strength of osteopenic rats. Better results might be achieved by prolonging the study period. Examination of the calcium homeostasis in the rats by isotopic methods might clarify the effects of annatto tocotrienol on calcium better. Nevertheless, this is the first study that examined the effects of skeletal biomechanical strength and bone calcium content in testosterone-deficient rats treated with annatto tocotrienol. 

## 5. Conclusions

In conclusion, annatto tocotrienol at 60 mg/kg for eight weeks is able to prevent mobilization of calcium from the bone into the circulation and increase skeletal calcium content. However, the dose and treatment should be adjusted to improve its effects on bone biomechanical strength. Further studies are warranted to justify a trial on the application of annatto tocotrienol in testosterone-deficient male osteoporotic patients.
